# Half Yearly Abstract of the Medical Sciences, &c. &c.

**Published:** 1845-10

**Authors:** 


*Half Yearly Abstract of the Medical Sciences, &c. &c.* Edited by Wm. H. Ranking, M. D., Cantab. Vol. 1. January—June, 1845. pp. 365. (Reprint.) J. & H. G. Langley: NewYork.

This is a publication on a somewhat similar plan to Braithwaite's Retrospect, over which it possesses advantages in the better selection and arrangement of its subjects, and especially in containing condensed *Reports* on the various branches of Medical Science. The Editor, Dr. Ranking, is an industrions contributor to the British Medical Journals, and, judging from his past performances, well qualified for the laborious task he has undertaken. We shall draw largely from the Abstract in future numbers of the Examiner.

